# Expression and clinicopathologic significance of TUFM and p53 for the normal–adenoma–carcinoma sequence in colorectal epithelia

**DOI:** 10.1186/s12957-017-1111-x

**Published:** 2017-04-27

**Authors:** Hong-Qing Xi, Ke-Cheng Zhang, Ji-Yang Li, Jian-Xin Cui, Po Zhao, Lin Chen

**Affiliations:** 1 Department of General Surgery, Chinese People’s Liberation Army General Hospital, 100853 Beijing, China; 2 Department of Pathology, Chinese People’s Liberation Army General Hospital, 100853 Beijing, China

**Keywords:** Colorectal carcinoma, Colorectal adenoma, TUFM, p53

## Abstract

**Background:**

Evidence indicates that most cases of colorectal carcinoma (CRC) develop from adenoma. A previous study demonstrated that mitochondrial Tu translation elongation factor (TUFM) might serve as an independent prognostic factor for colorectal cancer. However, the expression and function of TUFM in the normal–adenoma–cancer sequence have not been reported. In this study, we investigated the clinicopathologic significance of TUFM and p53 expression for the normal–adenoma–carcinoma sequence in colorectal epithelia and evaluated the roles of TUFM during the progression of colorectal tumors.

**Methods:**

Paraffin-embedded specimens from 261 colorectal normal mucosa samples, 157 adenomas, and 104 early carcinomas were analyzed for TUFM and p53 expression by immunohistochemistry.

**Results:**

Expression of TUFM and p53 was significantly increased during the colorectal normal–adenoma–carcinoma sequence (all *P* < 0.05). The expression of TUFM and p53 was associated with histologic type of adenomas (*P* = 0.028; *P* = 0.001) and grade of dysplasia (all *P* = 0.001). Expression of TUFM was positively correlated with that of p53 (*r* = 0.319, *P* = 0.001).

**Conclusions:**

Upregulated TUFM expression may play an important role in the transformation from colorectal normal mucosa to carcinoma through adenoma. Combined immunohistochemical detection of TUFM and p53 may be useful for evaluating the biological behavior of colorectal adenoma.

## Background

Colorectal adenoma is quite prevalent. Morson et al. [1, 2] suggested that most colorectal cancers evolve through the normal–polyp–cancer sequence. Colorectal adenoma is considered to be a precancerous state of colorectal cancer, and a previous study reported that the genetic alterations of colorectal adenoma were similar to those of colorectal carcinoma (CRC) [3].

Mitochondrial Tu translation elongation factor (TUFM), also called elongation factor Tu (EF-Tu), is one of the most abundant proteins of the mitochondria and participates in mitochondrial polypeptide biosynthesis [4]. TUFM has been studied as a key molecule that delivers aminoacyl-tRNA to the A-site on the ribosome during the process of protein translation in mitochondria [5]. In addition, other functions and characteristics have been reported for TUFM, including cell morphology and transformation, organization of mitotic apparatus, developmental regulation, cytokine response, and augmentation of autophagy [6–9]. However, TUFM’s biological role in the tumorigenesis process is unclear. Increasing evidence has indicated that dysregulation of TUFM is involved in the oncogenic process in various tumors. Upregulation of TUFM has been reported in lung [10], esophageal [11], gastric [12], liver [13], and pancreatic [14] cancers. A recent study showed that TUFM knockdown could lead to the induction of epithelial-mesenchymal transition (EMT) and increase metastasis and migration of A549 lung cancer cells [15]. Shi et al. reported that TUFM was overexpressed in CRC and further demonstrated that it could serve as an independent prognostic factor [16]. Evidence has indicated that most cases of CRC develop from adenoma [17]. We proposed that TUFM might play a role in the colorectal normal–adenoma–cancer sequence. To investigate this, the expression and clinicopathologic significance of TUFM in these different stages (normal mucosa, adenoma, and cancer) must be investigated. However, such studies have not been carried out.

The *TP53* gene is located on the short arm of chromosome 17p (17p13.1) and encodes a 53-kDa nuclear protein [18]. Wild-type *TP53* is a tumor suppressor gene [19], and its product (p53 protein) plays an important role in cell cycle arrest and induction of apoptosis [20, 21]. Mutant p53 is the most common abnormality in numerous tumor types, including colorectal cancer [22], and is associated with poor survival of colorectal cancer patients [23, 24]. Compared with wild-type p53, the mutant protein has no tumor growth-suppressing function but plays a role in increasing cell proliferation and enhancing susceptibility to tumor formation [25, 26]. Sui et al. reported that p53 could control cell invasion by inhibiting the NF-κB-mediated activation of Fascin [27]. Serum anti-p53 antibody was proved to be a useful biomarker for colorectal cancer screening [28]. p53 also plays critical roles in the transition from colorectal adenoma to carcinoma [29].

In our study, we evaluated changes in TUFM and p53 expression in the transition from adenoma to carcinoma and analyzed the association of TUFM and p53 expression in adenoma with clinicopathologic features. The relationship between TUFM and p53 in the colorectal normal–adenoma–carcinoma sequence was also explored.

## Methods

### Specimens

A total of 522 formalin-fixed and paraffin-embedded colorectal tissue specimens (104 early carcinomas, 157 adenomas, and 261 distal normal mucosa tissues paired with early carcinoma and adenoma) were obtained from the Department of Pathology, Chinese People’s Liberation Army (PLA) General Hospital. Ethical approval for this study was not required by our institution as the experiments carried out did not relate to patients’ privacy, impairment, or treatment. All of the early carcinomas were confined to the mucosa or submucosa. All specimens were from inpatients undergoing surgical operation from January 2006 to December 2010, and none of the patients received radiotherapy and chemotherapy before surgery. The age of early cancer patients ranged from 32 to 80 years (median 63 years; mean 62.6 ± 9.2 years) and that of adenoma patients ranged from 27 to 78 years (median 58 years; mean 57.3 ± 11.9 years). Patients’ clinical records and histopathology of each specimen were reviewed.

### Immunohistochemical analysis

Sections were cut at 1- to 4-μm thickness from paraffin blocks and mounted on 3-aminopropyltriethoxysilane (APES)-coated glass slides. After deparaffinization in xylene and rehydration through graded ethanol, the slides were heated in 0.01 mol/L citrate buffer (pH 6.0) in a microwave oven for 2 min and 30 s at 100 °C to retrieve antigens. Endogenous peroxidase activity was inhibited with 3% hydrogen peroxide for 15 min. After washing with phosphate-buffered saline (PBS), and blocking with 10% goat serum, the slides were incubated at 4 °C overnight with primary polyclonal rabbit anti-TUFM antibody (Abcam, Cambridge, MA) diluted 1:100 in blocking solution and monoclonal rabbit antibody to human p53 (Zymed, San Francisco, CA) diluted 1:80 in blocking solution. After washing with PBS, the sections were incubated for 30 min with polyperoxidase anti-mouse/rabbit IgG (Zymed). After rinsing in PBS, 3,3′-diaminobenzidine (DAB) (Zymed) was added as the chromogen. Finally, the slides were counterstained with hematoxylin. For the negative control, the primary antibody was replaced by PBS.

### Evaluation of immunohistochemistry

Evaluation of TUFM and p53 staining was carried out independently by two pathologists who were unaware of all clinicopathologic information. The whole section was screened for the expression of TUFM under light microscopy. The intensity of TUFM staining was scored as follows: 0, no staining; 1+, weak staining; 2+, moderate staining; and 3+, intense staining. The proportion of stained cells was scored as follows: 0, no cells stained; 1+, positive staining in <10% of cells; 2+, positive staining in 10–50% of cells; and 3+, >50% of cells stained positive. The final score was determined by the combined staining score. A score (extent + intensity) ≤1 was considered negative, and a score between 2 and 6 was considered positive [30, 31]. p53 staining was semiquantitatively assessed in ten representative fields chosen randomly under a microscope with a high-power (×400) objective. A total of 100 cells per field were scored. The specimens were considered positive when more than 10% of the neoplastic cells were immunoreactive [32]. In the case of discordant results, the sections were reevaluated on a multiheaded microscope.

### Statistical analysis

SPSS V.17.0 was used for statistical analysis. The Pearson chi-squared test was used to examine various clinicopathologic characteristics according to the expression of TUFM and p53. The Spearman’s correlation coefficient test was used to assess the association between the expression of TUFM and p53. A value of *P* < 0.05 was considered statistically significant.

## Results

### TUFM and p53 expression in the colorectal normal–adenoma–carcinoma sequence

TUFM protein was scored as positive in 22.2% (58/261) of normal mucosa, 42.0% (66/157) of adenoma, and 72.1% (75/104) of carcinoma tissues. There was a gradual increase in the TUFM-positive rate from normal mucosa to carcinoma through adenoma (all *P* < 0.05; Table 1).

**Table 1:** TUFM expression in colorectal normal mucosa, adenoma, and early carcinoma

Histology	TUFM		*P* value
	+	−	
Normal mucosa	58	261	*P = 0.001
Adenoma	66	91	**P = 0.001
Early carcinoma	75	29	***P = 0.001

*P* < 0.05, statistically significant

*P normal mucosa vs. adenoma, **P adenoma vs. early carcinoma ***P normal mucosa vs. early carcinoma

**Fig. 1:**
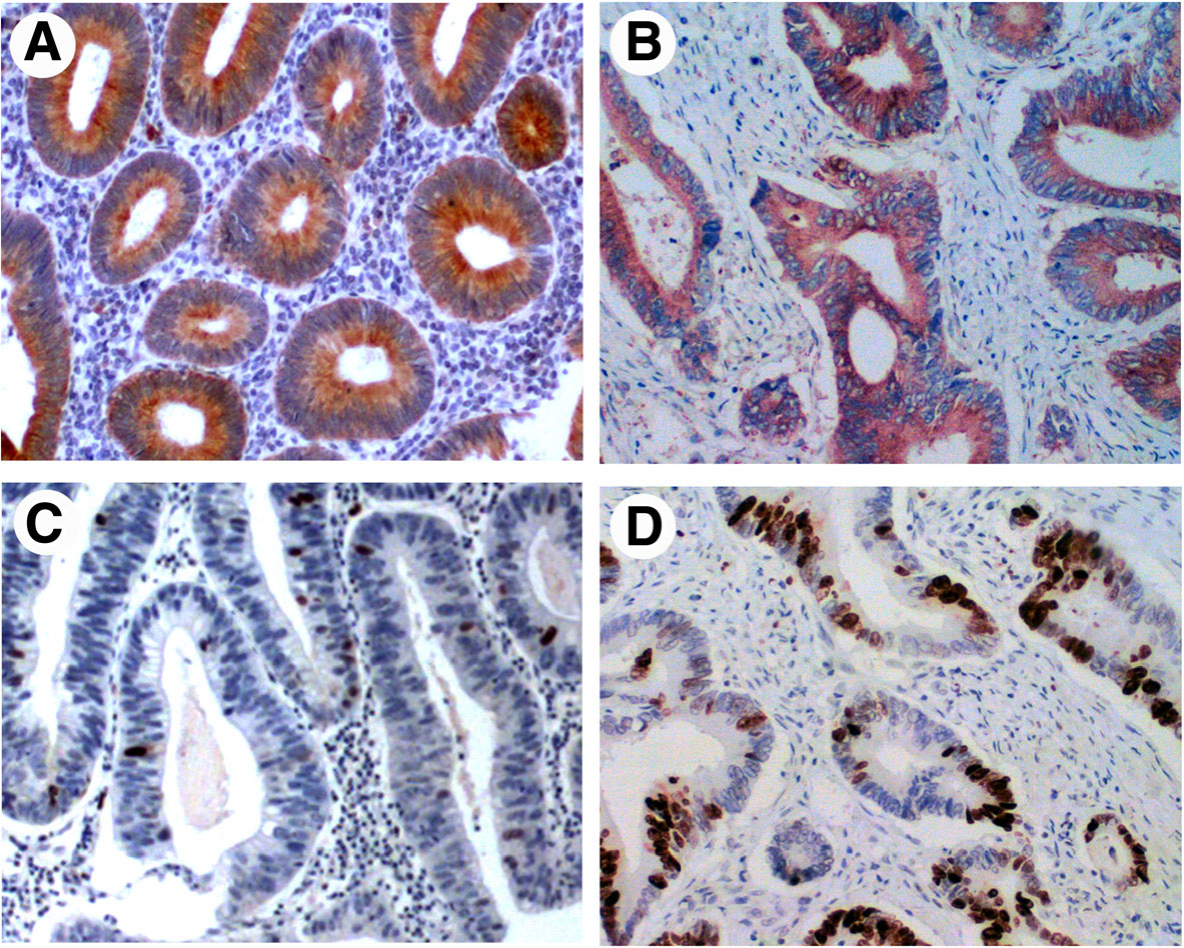
Representative immunohistochemical staining for TUFM and p53 in adenoma and colorectal carcinoma tissue. TUFM staining was positive in adenoma (**a**) (predominantly localized in the membrane and cytoplasm around the lumen of gland) and in carcinoma (**b**). Several scattered cells of moderate adenomas displaying p53 staining (**c**). Carcinoma shows positive staining for p53 (**d**)

Expression of TUFM protein was found primarily in the membrane and cytoplasm of adenoma or carcinoma cells (Fig. 1). p53 protein expression was not demonstrated in normal mucosa, but nuclear staining was detected in 22.3% (35/157) of adenomas and 60.6% (63/104) of carcinomas. The difference in p53 immunostaining between adenoma and carcinoma was significant (*P* = 0.001).

### Association of TUFM and p53 expression with clinicopathologic features in colorectal adenoma

Upon statistical analysis, a significant difference was found in TUFM expression according to histologic type of adenomas; TUFM showed a trend of more frequent positivity in tubulovillous adenoma (60%) compared with the other three types of adenoma (tubular, 33.3%; villous, 57.7%; serrated, 55.6%; *P* = 0.028). With respect to the grade of the adenoma, the positive rate of TUFM expression in adenomas with severe dysplasia was much higher than that in adenomas with mild and moderate dysplasia (*P* = 0.001; Table 2).

**Table 2:** Relationship between TUFM and p53 expression and clinicopathological parameters in colorectal adenomas

Variables	TUFM		*P* value	p53		*P* value
	+	−		+	−	
Gender						
Male	46	68	*P* = 0.486	26	88	*P* = 0.801
Female	20	23		9	34	
Age (years)						
<45	9	13	*P* = 0.923	4	18	*P* = 0.717
45 ≤ *n* < 60	26	33		12	47	
*n* ≥ 60	31	45		19	57	
Tumor size (cm)						
*d* < 1 cm	39	64	*P* = 0.083	16	87	*P* = 0.001
1 cm ≤ *d* < 2 cm	13	19		6	26	
*d* ≥ 2 cm	14	8		13	9	
Number of tumor						
*n* = 1	45	67	*P* = 0.456	24	88	*P* = 0.681
*n* ≥ 2	21	24		11	34	
Location						
Left side^a^	35	58	*P* = 0.115	21	72	*P* = 0.883
Right side^b^	20	27		11	36	
Both sides	11	6		3	14	
Histological type						
Tubular	34	68	*P* = 0.028	14	88	*P* = 0.001
Villous	15	11		14	12	
Tubulovillous	12	8		5	15	
Serrated	5	4		2	7	
Dysplasia						
Mild	28	64	*P* = 0.001	0	92	*P* = 0.001
Moderate	7	11		3	15	
Severe	31	16		32	15	

*P* < 0.05, statistically significant

^a^The left side comprises the splenic flexture, descending and sigmoid colon, and rectum

^b^The right side comprises the cecum, ascending colon, hepatic flexure, and transverse colon

**Fig. 2:**
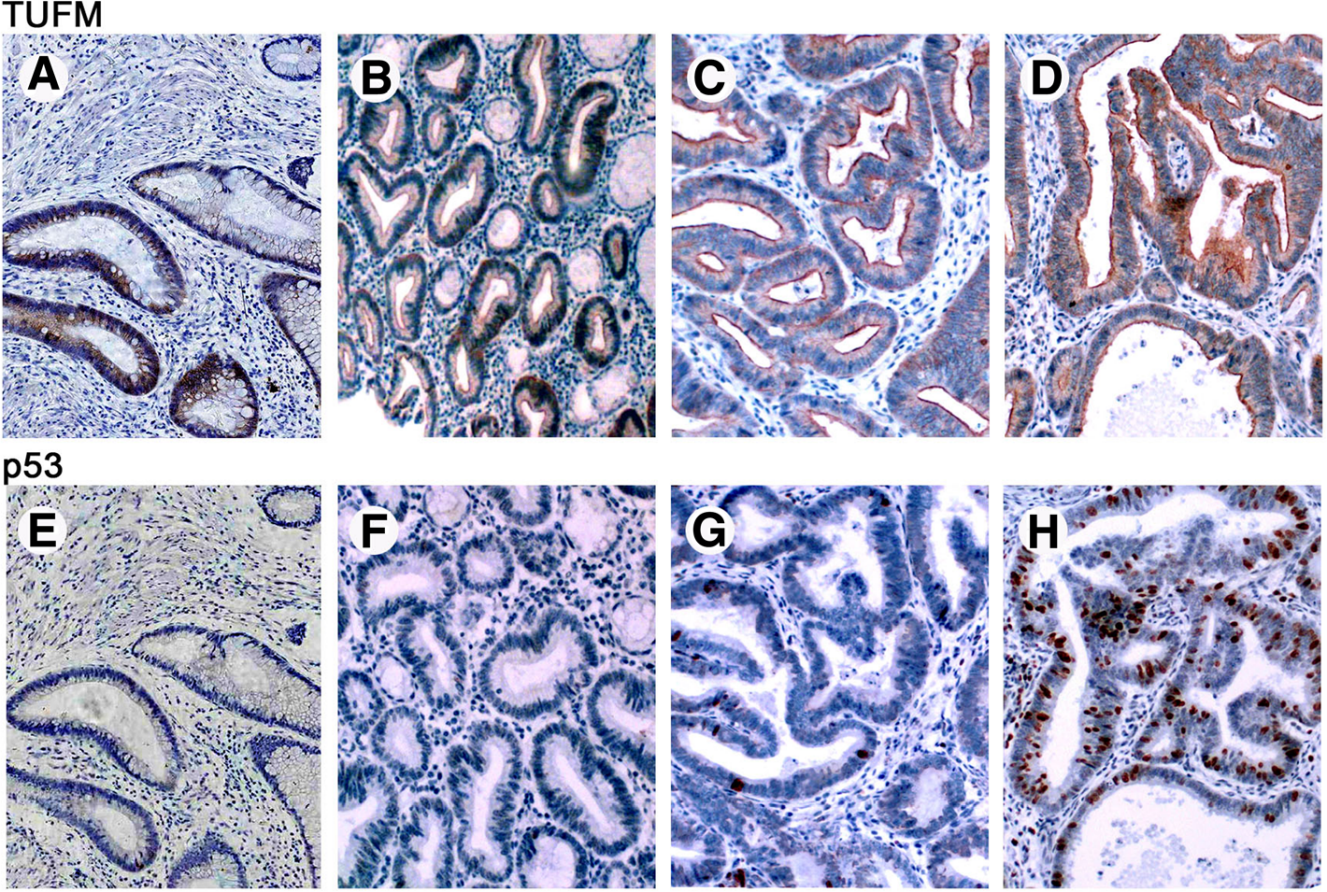
Immunohistochemical staining for TUFM and p53 in tissues representing the colorectal adenoma–carcinoma sequence. **a**–**d** TUFM was absent from colorectal normal mucosa and showed positive expression in lesions of different grades of dysplasia. **e**, **f** Normal colorectal mucosa and mildly dysplastic adenoma displaying negative p53 staining. **g** Several scattered cells of moderate adenomas displaying p53 staining. **h** Severe dysplasia adenoma or adenoma with cancer transformation exhibiting intense immunopositivity for p53 (**a**–**h**; ×100)

p53 expression was related to adenoma size (*P* = 0.001) and varied with different histologic types of adenoma (*P* = 0.001). p53 expression appeared to increase with advancing grade of dysplasia in adenoma (*P* = 0.001). p53 protein was absent in all mildly dysplastic adenoma samples. In adenomas with moderate dysplasia, p53 expression was found in several scattered cells. Adenomas with severe dysplasia showed intense nuclear expression of p52 and a higher level of p53 protein expression than adenomas with mild or moderate dysplasia (Fig. 2).

### Correlation between TUFM and p53 expression in adenomas

The Spearman analysis showed a significant positive correlation between TUFM and p53 expression in colorectal adenomas (*r* = 0.319, *P* = 0.001; Table 3).

**Table 3:** Correlation between TUFM and p53 expression in colorectal adenomas

		TUFM		Total
		+	−	
p53	+	25	10	35
	−	41	81	122
Total		66	91	157

## Discussion

Mitochondrial dysfunction was found to be associated with colon cancer progression [33]. Shi et al. showed that the level of TUFM overexpression could be considered an indicator of the degree of mitochondria dysfunction [16] and hypothesized that upregulation of TUFM protein is related to colorectal cancer progression. Additionally, Shi et al. have indicated that increased expression of TUFM in colorectal cancer is related to a poor clinical outcome and that TUFM could serve as a prognostic factor for cancer patients.

However, no investigation of TUFM expression and function has been performed in colorectal adenoma. In the current study, we demonstrated for the first time that the TUFM positive rate gradually increased from normal mucosa through adenoma to carcinoma (the normal–adenoma–cancer sequence) and was associated with the grade of dysplasia. The positive rate of TUFM expression in adenomas with severe dysplasia or with cancer transformation was much higher than that in adenomas with mild-grade dysplasia. This indicated that TUFM expression was more frequent in subjects likely to develop colorectal adenoma or carcinoma. We therefore presume that colorectal adenoma-to-carcinoma transformation may start in cells that already express TUFM and that TUFM may play important roles in the carcinogenesis and progression of colorectal cancer.

The results of our study indicated that TUFM showed a cytoplasmic staining pattern, which was consistent with its mitochondrial localization and similar to previous findings of Shi et al. [16]. Our results also showed that TUFM plays an important role in colon tumor progression. Therefore, TUFM-positive cells may promote the transition from adenoma to carcinoma. These findings could also explain why TUFM expression was present at a higher frequency in larger adenomas. Selective inhibition of TUFM in tumor cells might lead to mitochondrial collapse; therefore, anti-TUFM antibody-drug conjugates may warrant further evaluation for their ability to destroy TUFM+ cells of colorectal adenoma with canceration and early cancer.

Wild-type p53 has a tumor growth-suppressing capacity [19]. In contrast, mutant p53 does not have this function. Mutation in *TP53* is considered to be the most common genetic aberration in many types of tumors, including colorectal cancer [22]. Previous studies demonstrated that mutant *TP53* promotes the malignant transformation of colorectal adenoma to cancer [34]. Mutant p53 protein is associated with increased cell proliferation and loss of the capacity for apoptosis induction [25, 26]. The wild-type p53 protein could not be detected by the immunohistochemical method because of its relatively short half-life; however, the mutant p53 protein has a longer half-life and greater stability and is immunohistochemically detectable [35, 36]. Approximately 50% of human primary tumors have *TP53* gene mutations [22, 37]. In our study, p53 was undetectable in normal colorectal mucosa and the positive rate of p53 in early carcinoma (61.7%) was significantly higher than that in adenoma (21.5%). These results are in agreement with previous reports [34, 38]. We showed that p53 expression began to be detected in a few scattered cells in adenomas with moderate dysplasia and showed intense nuclear staining in adenomas with severe dysplasia and early carcinomas. This supports the notion that p53 mutation occurs at a moderately dysplastic stage during conversion from adenoma to early cancer [34, 39].

Screening for adenomas and early-stage CRC has decreased the incidence and mortality for CRC in the USA over the past few decades [40]. However, current screening methods do not provide adequate sensitivity and efforts are continuing to be directed toward the development of novel diagnostic or screening serum markers for CRC. One of the most important findings in this study is the positive correlation between TUFM and p53 expression in colorectal adenoma. This association indicated that upregulated TUFM expression during the colorectal normal–adenoma–carcinoma sequence may contribute to the transformation from normal mucosa to carcinoma through adenoma. We presumed that TUFM overexpression, as an early event in colorectal tumorigenesis, may take place before p53 mutations occur because TUFM expression appeared earlier than p53 (in the mild-grade stage versus the moderate stage) in the colorectal normal–adenoma–carcinoma sequence.

## Conclusions

In conclusion, upregulated TUFM expression may play an important role in the transformation from colorectal normal mucosa to carcinoma through adenoma. Combined immunohistochemical detection of TUFM and p53 could, to some extent, reflect the biological behavior of colorectal adenoma and might be helpful in differential diagnosis of benign adenoma and adenoma with early cancer potential, which is sometimes difficult depending on the histologic examination or hematoxylin-eosin staining. However, further extensive studies are necessary to determine and clarify the contribution of TUFM to the malignant transformation from adenoma to carcinoma and guide clinical diagnosis and treatment of colorectal adenoma and carcinoma.

## Abbreviations

CRC: Colorectal carcinoma
PBS: Phosphate-buffered saline
TUFM: Mitochondrial Tu translation elongation factor
